# Does insecticide resistance contribute to heterogeneities in malaria transmission in The Gambia?

**DOI:** 10.1186/s12936-016-1203-z

**Published:** 2016-03-15

**Authors:** Kevin Ochieng’ Opondo, David Weetman, Musa Jawara, Mathurin Diatta, Amfaal Fofana, Florence Crombe, Julia Mwesigwa, Umberto D’Alessandro, Martin James Donnelly

**Affiliations:** Medical Research Council Unit, PO Box 273, Banjul, The Gambia; Department of Vector Biology, Liverpool School of Tropical Medicine, Liverpool, UK; London School of Hygiene and Tropical Medicine, London, UK; Institute of Tropical Medicine, Antwerp, Belgium

## Abstract

**Background:**

Malaria hotspots, areas with consistently higher than average transmission, may become increasingly common as malaria declines. This phenomenon, currently observed in The Gambia, may be caused by several factors, including some related to the local vectors, whose contribution is poorly understood.

**Methods:**

Using WHO susceptibility bioassays, insecticide resistance status was determined in vector populations sampled from six pairs of villages across The Gambia, each pair contained a low and high prevalence village.

**Results:**

Three vector species were observed (23.5 % *Anopheles arabiensis*, 31.2 % *Anopheles gambiae*, 43.3 % *Anopheles coluzzii* and 2.0 % *An. coluzzii* × *An. gambiae* hybrids). Even at a fine scale, significant differences in species composition were detected within village pairs. Resistance to both DDT and deltamethrin was more common in *An. gambiae*, most markedly in the eastern part of The Gambia and partly attributable to differing frequencies of resistance mutations. The *Vgsc*-*1014F* target site mutation was strongly associated with both DDT (OR = 256.7, (95 % CI 48.6–6374.3, p < 0.001) and deltamethrin survival (OR = 9.14, (95 % CI 4.24–21.4, p < 0.001). A second target site mutation, *Vgsc*-*1575Y*, which co-occurs with *Vgsc*-*1014F*, and a metabolic marker of resistance, *Gste2*-*114T,* conferred additional survival benefits to both insecticides. DDT resistance occurred significantly more frequently in villages with high malaria prevalence (p = 0.025) though this did not apply to deltamethrin resistance.

**Conclusion:**

Whilst causality of relationships requires further investigation, variation in vector species and insecticide resistance in The Gambia is associated with malaria endemicity; with a notably higher prevalence of infection and insecticide resistance in the east of the country. In areas with heterogeneous malaria transmission, the role of the vector should be investigated to guide malaria control interventions.

**Electronic supplementary material:**

The online version of this article (doi:10.1186/s12936-016-1203-z) contains supplementary material, which is available to authorized users.

## Background

Malaria foci, referred to as ‘hot spots’, have persistently higher transmission rates [1–3] than contiguous areas and pose challenges to malaria control programmes. They may be refractory to conventional malaria control tools and may act as sources of infection to surrounding areas [4, 5]. As transmission falls, partly in response to control scale-up, [6] heterogeneity in transmission will become more apparent [7, 8]. Marked heterogeneity in transmission has been documented [9, 10] even at the village level [11, 12], and in areas of overall reduced transmission like The Gambia [13, 14].

Understanding the epidemiological factors that contribute to the emergence and maintenance of these hotspots is crucial for malaria elimination. Human [15–17] and vector behaviour [18, 19], environmental factors [20–23] and their interplay may give an insight into the transmission dynamics in hotspots. Malaria vector species and populations vary in space and time [18], in anthropophily, exophily and endophily [19] and, importantly, in insecticide susceptibility [24].

Resistance to available insecticides has been widely reported in malaria vectors [25–31]. Although a causal relationship between insecticide resistance and malaria transmission has not been shown, spatial variation in susceptibility to insecticides is likely to contribute to the observed heterogeneity in malaria transmission [32]. Since mosquitoes resistant to insecticides survive longer than their susceptible counterparts in the presence of an insecticide, they may live long enough [33] to affect malaria transmission [34–36]. Therefore, insecticide-resistant vectors may maintain transmission [37] or, where control interventions have been successful, reverse gains [35, 38, 39].

### Malaria in The Gambia

In The Gambia, malaria transmission has decreased substantially over the last few years and has become increasingly heterogeneous [6, 13, 14]. Malaria transmission follows rainfall, beginning after the onset of the rains and peaking between October and November. Malaria prevalence in children under the age of 5 years is nationally 4–5 %, though in some areas between 2 and 15 % [6, 14, 40]. In the eastern Gambia, cross-sectional survey across all ages in 2012 estimated malaria prevalence at above 30 %.

Malaria control, coordinated by the Gambia National Malaria Control Programme (GNMCP), largely employs long-lasting insecticide-treated bed nets (LLINs) and indoor residual spraying (IRS) with DDT [41]. Between 2013 and 2014, the GNMCP carried out a mass LLIN distribution campaign with Permanet^®^. While a cross-sectional survey across Gambian villages showed over 90 % bed net use in 2012 [13], the National LLIN usage in children under the age of 5 years stands at 60 % while in pregnant women it is only 40 % [14]. Annual IRS with DDT has been done since 2008 throughout the country except the coastal region where malaria transmission is extremely low. The first-line treatment is artemether-lumefantrine; pregnant women receive sulfadoxine-pyrimethamine as intermittent preventive treatment while children 3–59 months old in upper and central river regions (URR and CRR) obtain seasonal malaria chemoprevention with amodiaquine and sulfadoxine-pyrimethamine since the 2014 transmission season.

Vector control activities carried out by GNMCP have probably played a major role in reducing transmission [14]. However, these gains may be reversed by insecticide resistance that has been recently observed in The Gambia [42, 43]. Vector species distribution varies from east to west along the River Gambia [44]. Four malaria vectors, *Anopheles gambiae s.s., Anopheles coluzzii, Anopheles arabiensis* and *Anopheles melas* maintain transmission. *Anopheles melas* is mainly confined to brackish waters near the coastal region but extends up to approximately 200 km inland during the rainy season [44–46]. During the rainy season, the population of *An. gambiae s.s.* rises non-uniformly across the country while *An. arabiensis* and *An. coluzzii* persist longer into the dry season [44].

The local dynamics of insecticide resistance may be impacted by the spatio-temporal variation in insect vectors [47–49], which can result from different ecological niche preferences [50, 51]. In scenarios where populations are separated by ecological factors or barriers, different resistance mechanisms may develop as a result of differential selection pressure or the occurrence of different mutations. Nonetheless, occasional gene flow [52] can transfer mutations [53–56] which may rise rapidly in frequency if selected by anthropogenic activity.

As part of a larger study investigating malaria transmission dynamics in The Gambia, the distribution and patterns of phenotypic resistance and mechanisms in *An. gambiae s.l.* populations was characterized. Specifically, the hypothesis that variation in the intensity of malaria transmission may be linked with variation in insecticide resistance, mediated by differences in species composition and resistance-related mutations was examined.

## Methods

### Study sites

**Fig. 1:**
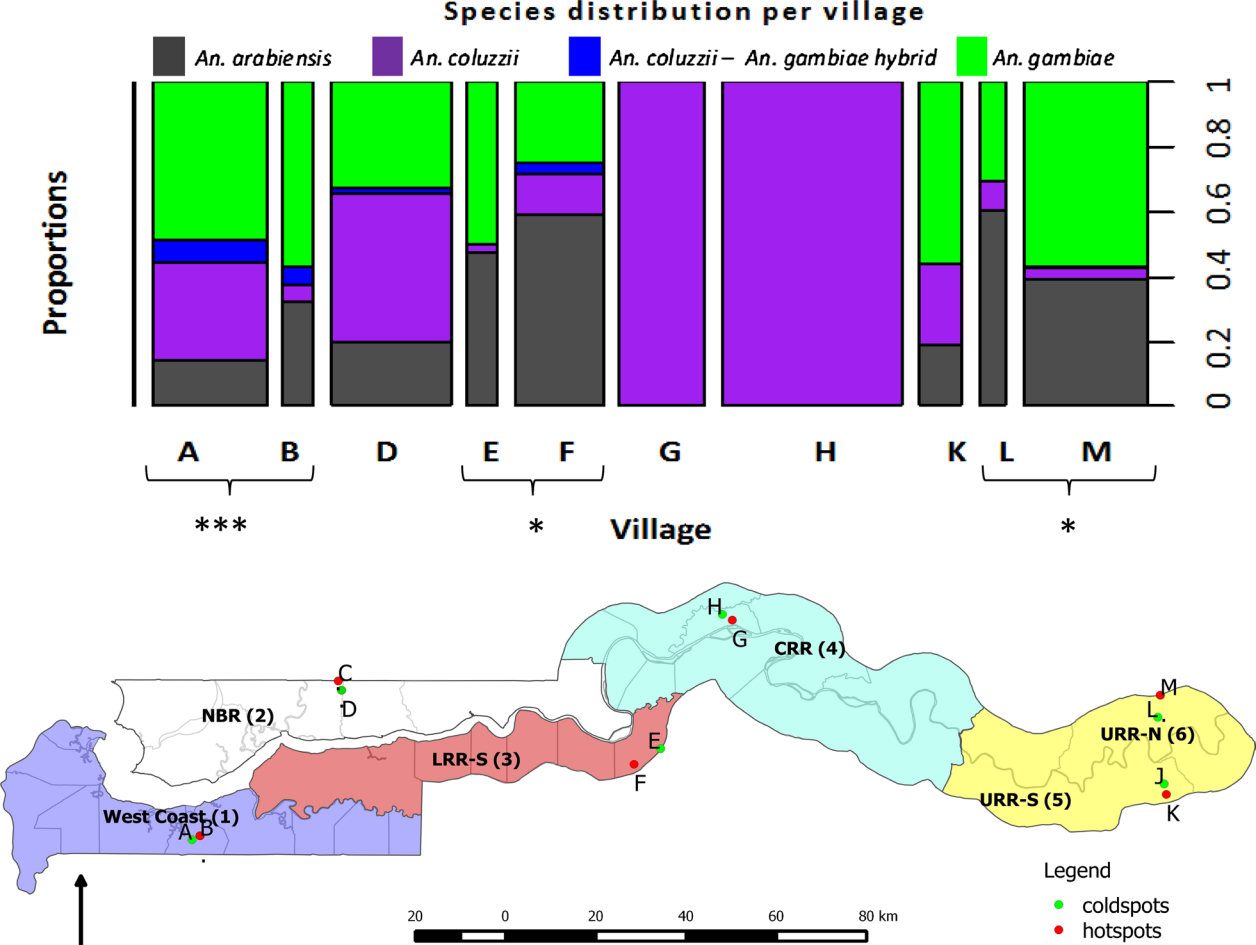
Graph showing the relative proportion of malaria vectors across study sites in The Gambia. *Asterisks* indicated significant differences in species diversity between village pairs assessed using fisher exact tests (where collections and > 1 species collected, permitted test); ***P < 0.001; *P < 0.05 Below it is administrative map of The Gambia showing study sites/villages in 6 geographic regions (numbered). *1* west coast, *2* LRR-N (lower river region-north), *3* LRR-S (lower river region-south), *4* CRR-N (central river region-north), *5/6* URR-S/N (upper river region-north/south). The *green dots* indicate low malaria prevalence while *red dots*—high malaria prevalence villages. *A* Bessi, *B* Ndemban Tenda, *C* Chogen Wellingara, *D* Yallal Ba, *E* Sinchu Njengudi, *F* Dongoro Ba, *G* Sare Seedy, *H* Ngedden, *K* Madina Samako, *J* Njaiyel, *L* Sare Wuro, *M* Gunjur Koto

The study was conducted in The Gambia, a West African country surrounded by Senegal except to the west that borders the Atlantic Ocean. The country is divided into five administrative regions, namely west coast, lower river region—south (LRR-south), lower river region—north (LRR-north), central river region (CRR) and upper river region (URR) (Fig. 1). For purposes of this study and the overall study investigating transmission dynamics, URR was subdivided into URR—north and south to form a total of six regions. Six pairs of rural villages, one pair per region, were selected on the basis of malaria prevalence determined by a nationwide cross-sectional survey [13] (Fig. 1). In each pair, the village with the highest prevalence and that with the lowest prevalence were included. For all pairs there was a significant difference in infection prevalence with the exception of villages G and H in the central river region (Additional file 1: Table S1).

The Gambia has one rainy season from June to October diminishing in November. The mean daily temperature varies between 25 and 40 °C. The country is primarily low lying with seasonal flooding; and is situated in the open and flat woodland Savannah belt and riverine swamps are common towards the western part of the country [44, 57]. The sea mixes with the river and during the rainy season, brackish waters can extend 200 km upstream. Rice paddies are common on the margins of the river, especially in the CRR. Towards the east, cereal crop farming is practised. Between the months of June and September 2013, coinciding with mosquito sampling, the GNMCP distributed LLINs and sprayed houses with DDT in the country including all our study villages with the villages in region 2 being sprayed last in the months of September/October.

### Study design

Mosquitoes were sampled between July and October 2013 from the 12 villages. Larval collections were conducted within a 2 km radius of the centre of the villages and transported to a central insectary in Wali Kunda (13°34′N, 14°55′W) for rearing and testing. Blood fed adult female collections were performed in villages that had few or no observable breeding habitats. Blood-fed anophelines were transferred to the insectary on the same day of collection where they were kept in individual paper cups containing moistened Whatman filter papers to induce egg laying. The females were also provided with 10 % glucose solution on a cotton wool plug. Eggs from blood-fed mosquitoes from one village were grouped together and allowed to mix randomly.

Mosquitoes, including an insecticide-susceptible colony from Yaoundé, Cameroon, were reared under similar conditions. Larvae were fed on Tetramin^®^ (Tetramin gmbH Germany) fish food and maintained at 28 °C and 80 % humidity. Upon emergence, adult mosquitoes were provided with 10 % glucose. The WHO protocol [58] on insecticide susceptibility tube assays was used to assay phenotypic resistance.

Three to five day old mosquitoes in groups of 20–25 were exposed for an hour to either 4 % DDT or 0.05 % deltamethrin impregnated papers [58]. These two insecticides were chosen because the GNMCP distributes deltamethrin-impregnated LLINs (Permanet^®^) and uses DDT in IRS campaigns. A total of 1005 field collected *An. gambiaes.l.* were tested. Mortality in the control group (susceptible colony from Yaoundé Cameroon) was always less than 5 %. After the phenotypic assays, all mosquitoes tested were stored in 1.5 ml Eppendorf tubes with silica gel and transported to the MRC Fajara for species identification and molecular screening of insecticide resistance loci.

### Laboratory processing

DNA from all mosquitoes was extracted using a Qiagen kit according to manufacturer’s protocol. Two polymerase chain reaction protocols [59, 60] were used to identify the *An. gambiae s.l.* to species level. The protocol of Scott et al. was used to identify *An. gambiae s.s., An. arabiensis, An. melas* while the SINE-PCR [60] protocol was used to further distinguish the *An. gambiae s.s.*, from *An. coluzzii* and *An. arabiensis,* simultaneously.

All mosquitoes tested in the insecticide resistance bioassay were genotyped, using TaqMan assays [61–64], for five markers of insecticide resistance, namely the *Vgsc*-*1014F* and *Vgsc*-*1014S* mutations in the voltage gated sodium channel gene that confer resistance to DDT/pyrethroids, *Vgsc*-*1575Y* which enhances action of the *1014F* mutation, *Gste2*-*114T* which has been associated with metabolic resistance to DDT, and *Ace1*-*119S* which is associated with resistance to carbamates and organophosphates [63].

### Statistics

Statistical analysis was done using R statistical package (R version 3.1.2, 2014). Tests of differences in proportions were done to investigate differences in vector populations. Fisher’s test was used to determine differences in species composition using an online algorithm. Pearson’s Chi squared test for proportions was used to test for differences in mortality between species and villages. Non-parametric tests were used to investigate differences in mortality to insecticides within pairs of study villages and, more generally, geographic variation in insecticide resistance. Differences between individual proportions were assessed using Marascuilo’s procedure [65].

Binomial confidence intervals [66, 67] were calculated for species distribution and mortality to insecticides. Odds ratios were used to estimate the effect size of DNA marker assays in relation to resistance phenotype. Further, general linear models (GLM) with logit link function for a binomial dependent variable was used to model the effect of different mutations, sampling site, species and interaction between DNA resistance markers on survivorship.

Differences in mortality trend was determined by first grouping villages into three regions, eastern, central and western villages according to ecological zones identified by Caputo et al. [44]. Western villages consisted of A: Bessi, B: Ndemban Tenda, C: Chogen Wellingara and D: Yallal Ba, central villages were E: Sinchu Njengudi, F: Dongoro Ba, G: Sare Seedy and H: Ngedden, and eastern villages were J: Njaiyel, K: Madina Samako, L: Sare Wuro and M: Gunjur Koto.

### Ethical clearance

This study was approved by Medical Research Council Unit (MRC) scientific coordinating committee and ethical clearance obtained from The Gambia Government/MRC Joint Ethics committee. Informed oral consent was obtained during village sensitization meetings.

## Results

**Fig. 2:**
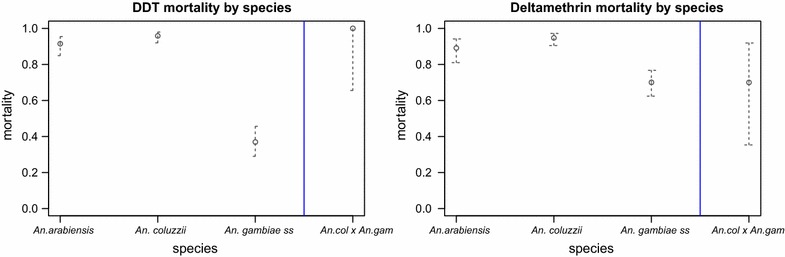
Species-specific mortality to DDT (*left*) and deltamethrin (*right*)

In the 2013 collection season, 1005 mosquitoes were tested using the WHO tube bioassay protocol [58]; 508 against 4 % DDT, 497 against 0.05 % deltamethrin. *An. gambiaes.l.* was sampled from all but two villages, Madina Samako and Chogen Wellingara (Fig. 2). Three members of the *An. gambiae* complex were identified: *An. gambiae s.s., An. arabiensis,* and *An coluzzii* together with some *An. gambiae* s.s. x *An. coluzzii* hybrids (Fig. 1 and Additional file 1: Tables S2, S3, S4). Of the paired study villages, vector composition could only be compared in four pairs because the two remaining pairs lacked mosquitoes in one or both of the constituent villages. In three of the four village pairs, species composition varied between high and low transmission village pairs (Fig. 1).

### Phenotypic resistance to DDT and deltamethrin in a WHO bioassay

There were significant interspecies differences in the 24 h post-exposure mortality to DDT and deltamethrin. For DDT, resistance was most pronounced in *An. gambiae s.s.*, with only 37 % mortality (95 % CI 29–46 %), compared to the other four species (Pearson Chi squared test, χ^2^ = 194, df = 3, p < 0.001) (Fig. 2). Further analysis showed significant differences in mortality except between *An. arabiensis* and *An. coluzzii* (Additional file 1: Tables S2, S3, S4). There were also significant differences in mortality between species following deltamethrin exposure (Pearson’s Chi squared test, χ^2^ = 44.94, df = 3, p < 0.001). A significant difference in mortality was only observed when species were compared to *An. gambiaes.s.,* with the exception of *An. coluzzii* × *An. gambiae s.s* hybrids (Additional file 1: Tables S2, S3, S4).

There was a significant correlation between DDT and deltamethrin mortality (Kendall’s correlation weighted by village, τ = 0.61, p = 0.02), indicating that, *An. gambiae s.s.* populations were likely to be resistant to both insecticides.

**Fig. 3:**
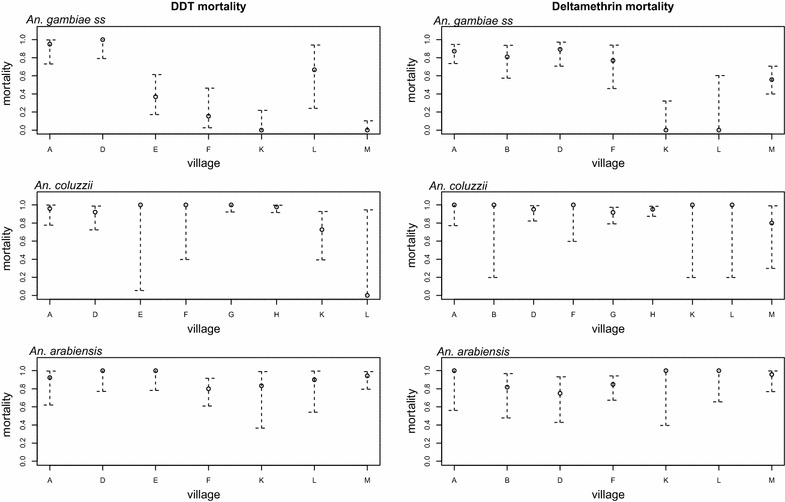
Mortality by species and village (sampling site). Mortality against DDT (*left*), mortality against deltamethrin (*right*). Villages are labelled as in Fig. 1

There was variability in inter species mortality within and between villages for deltamethrin (χ^2^ = 9.14, p = 0.03) and DDT (χ^2^ = 7.78, p = 0.05). *An. gambiae s.s.* from the east were more resistant than those from the western part of the country (Table 1). DDT mortality tended to decrease from west to east, starting from Sinchu Njengudi (E). For deltamethrin, there was a similar trend though reduced mortality was mainly in Madina Samako (K), Sare Wuro (L) and Gunjur Koto (M) (Fig. 3).

**Table 1:** Differences in *Anopheles gambiae s.l.* mortality between east and western populations

Insecticide	Species	Region	Mortality (%)	χ^2^	Df	P
DDT	*An. gambiae s.s.*	East	6	82.42	1	<0.001
		West	97			
	*An. arabiensis*	East	92	0.11	1	0.74
		West	97			
	*An. coluzzii*	East	67	4.75	1	0.03
		West	94			
Deltamethrin	*An. gambiae s.s.*	East	41	32.56	1	<0.001
		West	86			
	*An. arabiensis*	East	97	2.55	1	0.11
		West	83			
	*An. coluzzii*	East	89	0.04	1	0.85
		West	97

### Resistance association of DNA markers

The frequency of resistance alleles for various markers varied among species, with the *Vgsc-1014F* mutation being most common in *An. gambiae s.s*. (Table 2), and in this species there was a highly significant association with resistance to both DDT and deltamethrin. It was not possible to conduct these tests on the other species due to the low frequency of the *Vgsc*-*1014F* resistance mutation and high mortality.

**Table 2:** Allele frequency, in percentage expressed as proportions (allele/total number of alleles), of insecticide resistance mutations of malaria vectors in The Gambia

Species	*Vgsc*-*1014F*	*Vgsc*-*1014S*	*Vgsc*-*1575Y*	*Gste2*-*114T*	*Ace1*-*119S*
Allele frequency (with 95 % confidence intervals) of molecular resistance markers by species					
* An. gambiae s.s.*	0.51 (0.45–0.55)	0.004 (8.56^−5^–0.01)	0.13 (0.1–0.16)	0.097 (0.075–0.12)	0.003 (5.68^−4^–0.01)
* An. arabiensis*	0.05 (0.02–0.06)	0.14 (0.1–0.17)	0.002 (1.13^−4^–0.01)	0.017 (0.008–0.04)	0 (0–0.1)
* An. coluzzii*	0.0012 (6.16^−5^–7.62^−3^)	0 (0–0.01)	0.0012 (6.16^−5^–0.01)	0.67 (0.67–0.74)	0 (0–0.01)
* An. coluzzii* × *An. gambiae* hybrid	0.15 (0.063–0.31)	0 (0–0.11)	0.08 (0.02–0.22)	0 (0–0.11)	0 (0–0.11)

In *An. gambiae s.s.,* survival of the *Gste2*-*114T* carriers was also significantly increased for DDT and (unexpectedly) for deltamethrin (Table 3). For *An. coluzzii,* there was no significant association between *Gste2*-*114T* and DDT resistance although a significant negative effect was observed for deltamethrin (Table 3).

**Table 3:** Odds ratios of *An. gambiae s.s.* and *An. coluzzii* mutants surviving an insecticide exposure for each insecticide resistance marker

Species	Insecticide	Marker	Odds ratio	95 % confidence intervals		P
				Lower	Upper	
*An. gambiae s.s*.	DDT	Kdr	253.74	48.07	6302.05	<0.001
		Gste2	3.4	1.43	9.18	0.01
	Deltamethrin	Kdr	8.37	3.99	18.47	<0.001
		Gste2	3.4	1.175	10.29	0.02
*An. coluzzii*	DDT	Gste2	1.5	0.34	11.35	0.72
	Deltamethrin	Gste2	0.23	0.06	0.78	0.02

Species, village and *Vgsc*-*1014F e*xplained significant variation in mortalities to both insecticides, though *Gste2*-*114T, Vgsc*-*1575Y* and interactions between markers were not significant (Table 4). Because of the absence of a sufficient number of survivors carrying resistance mutations in other species, other than *An. gambiae s.s.,* interaction between species and markers was not included in the model. A backward stepwise logistic regression therefore excluded *Gste2*-*114T* and *Vgsc*-*1575Y* in the final model (Additional file 1: Tables S3, S4). All the molecular markers screened in this study played a role in insecticide resistance but their effect was masked by the presence of the *Vgsc*-*1014F* mutation in captured *An. gambiae* s.s. which was a strong predictor of insecticide resistance.

**Table 4:** The effects of village, species and resistance markers on mortality of mosquitoes to DDT and deltamethrin using GLM

Factor	Df	Deviance	Residual Df	Residual deviance	P
DDT					
Species	5	190.56	490	331.83	<0.001
Village	8	114.2	482	217.62	<0.001
*Kdr*	5	51.04	477	166.58	<0.001
*1575y*	2	1.05	475	165.53	0.59
*Gste2*	2	1.8	473	163.73	0.41
*Kdr:1575Y*	1	0.86	472	162.87	0.35
*kdr:gste2*	4	4.33	468	158.55	0.36
*1575Y:gste2*	2	1.13E−08	466	158.55	1
Deltamethrin					
Species	5	50.46	482	371.69	<0.001
Village	8	34.45	474	337.24	<0.001
*Kdr*	5	28.22	469	309.02	<0.001
*1575y*	2	3.13	467	305.9	0.21
*Gste2*	2	4.26	465	301.63	0.12
*Kdr:1575Y*	1	0.09	464	301.54	0.76
*Kdr:gste2*	3	4.07	461	297.47	0.25
*1575Y:gste2*	3	0.86	458	296.61	0.83

### Insecticide resistance and malaria transmission

For *An. gambiaes.s.,* mortality to DDT and deltamethrin was compared between high and low malaria prevalence villages. Data from the only village pair where there was no apparent difference in malaria infection rates (p = 0.08; villages G and H, central river region) are excluded as *An. coluzzii* was the only species collected. The unpaired Wilcoxon sum rank test was used because some villages did not have mortality data. DDT mortality for *An. gambiaes.s.* was significantly lower in high prevalence than low prevalence villages (Wilcoxon W = 0, p = 0.03). There was no observed difference in *An. gambiae s.s.* mortality to deltamethrin between high and low prevalence villages (W = 3.5, p = 0.24) or for any of the other species for both insecticides.

## Discussion

Phenotypic resistance to DDT and deltamethrin was found mainly in *An. gambiae s.s.* and was more common in eastern Gambia where malaria transmission is higher than in the western regions [13, 68, 69], suggesting a link between insecticide resistance and observed malaria prevalence. Previous studies exploring the association between insecticide resistance and malaria endemicity have produced contrasting results, with some reporting no effect [70–73] while others suggesting otherwise [32]. In neighbouring Senegal [74] and in South Africa [35], following successful malaria control, increasing insecticide resistance coincided with higher incidence of clinical malaria. Nevertheless, proving a causal relationship between insecticide resistance and malaria transmission is extremely difficult [75].

Similar to earlier studies [68, 76, 77], three malaria vectors, namely *An. gambiae s.s., An. coluzzii* and *An. arabiensis*, were observed across the country and in different proportions, in addition to a few hybrids of *An. gambiae s.s.* and *An. coluzzii*. *Anopheles melas*, known to breed in brackish water and usually found in western Gambia [44, 45, 78], was not collected. This may have been due to the rearing methods employed in the insectary.

The extreme interspecific differences observed in insecticide resistance status and frequency of mutations among them suggests that the involvement of insecticide resistance in malaria heterogeneity would be conditional on the vector species composition. This may help explain the differences in insecticide susceptibility estimates reported by two previous studies in eastern Gambia. In one study done in 2010, [43], *An gambiae s.l.* susceptibility to DDT and pyrethroids was about 90 % while in 2011 in a village of the same region, susceptibility to the same insecticides was only 50 % [42]. Such differences may be explained by the composition of the mosquito population tested. Indeed, in 2010, 70 % of all anophelines were *An. arabiensis,* while in 2011 this species represented only 42 % of all mosquitoes tested. Therefore, the high proportion of *An. arabiensis* may have concealed resistance in *An. gambiae s.s*.

### Mechanisms of resistance

In *An. gambiae s.s.,* there was a clear association between the *Vgsc*-*1014F* mutation and phenotypic resistance, indicating that in The Gambia this is a very effective predictor of DDT and pyrethroids resistance. The *Vgsc*-*1575Y* and *Gste2*-*114T* markers had modest effects in conferring phenotypic resistance. Though in *An. gambiae s.s* and *An. arabiensis* the *Vgsc*-*1014S* mutation did not seem to be linked to phenotypic resistance, its low frequency limited statistical power. As in Uganda [79], few samples had both serine and phenylalanine mutations though carriers were also resistant to DDT. Given the low frequency of co-occurrence, it is not possible to establish whether carriage of both mutations confer an advantage, though this may be the case, at least compared to serine alone [80].

### Population subdivision

The different insecticide resistance profile between eastern and western Gambia raises important questions about the drivers and stability of this heterogeneity. The GNMCP has distributed LLINs across the country since 2003 and sprayed houses yearly with DDT since 2008 [14], though only intermittently in the urban west coast region because of the lower malaria transmission. IRS has been carried out in all study villages so that DDT selection pressure should have been uniform. Nevertheless, intense DDT use in a community trial investigating the additional benefits of IRS with DDT to LLIN may have increased insecticide resistance pressure [43, 81].

With no history of carbamate and/or organophosphate use for public health in The Gambia, it is interesting to note that the two mosquitoes that had a carbamate/organophosphate resistance allele, *Ace1-119S,* were sampled from a village that is approximately 70 km from Guinguineo district, Senegal, where resistance to bendiocarb has been reported [82, 83], (President’s Malaria Initiative, Senegal Report, unpublished), possibly linked to intense IRS campaigns with bendiocarb between 2008 and 2013. Investigation on the genetic connectivity between Gambian and Senegalese *An. gambiae* populations is currently underway.

Host seeking/foraging and resting behaviour of mosquitoes have been shown to play a role in the development of insecticide resistance [84, 85]. In The Gambia, the lack of detailed information on the behaviour of the sympatric malaria vectors limits proper insights into the causes of resistance in the eastern populations. Endophagy of *An. gambiae s.s.* may increase their exposure to insecticides, favouring the development of resistance [86, 87]. Conversely, exophagy of *An. arabiensis* [77] could play a role in the low levels of resistance observed in this species. However, in Senegal, where no difference in biting and host seeking behaviour were found [88] until recently [89], resistance has been reported mainly in *An. gambiae s.s.* and to a lesser extent in *An. arabiensis* and *An. coluzzii* [24, 89].

## Conclusion

Insecticide resistance, which varies by species, seems to be associated to malaria endemicity although other factors not studied here may also be involved. Indeed, in eastern Gambia both insecticide resistance and malaria transmission are higher than in the rest of the country. The vector population is also extremely heterogeneous, underpinning the need for national malaria control programmes to continually monitor, as extensively as possible, the status of insecticide resistance to guide malaria control practices.

## Additional file

10.1186/s12936-016-1203-z Comparison of malaria prevalence rates between villages. **Table S2, S3, S4.** Statistical analysis of species specific mortality to DDT and deltamethrin.
