# Supplementary material for: Does insecticide resistance contribute to heterogeneities in malaria transmission in The Gambia?
Source: Malar J. 2016 Mar 15;15:166. doi: 10.1186/s12936-016-1203-z (PMC4793517; doi:10.1186/s12936-016-1203-z)
Supplement: Supplementary file 1 — 10.1186/s12936-016-1203-z Comparison of malaria prevalence rates between villages. Table S2, S3, S4. Statistical analysis of species specific mortality to DDT and deltamethrin. [file 12936_2016_1203_MOESM1_ESM.docx]

Table 1: Prevalence of asymptomatic *Plasmodium falciparum* as determined by PCR by site and village in 2012 cross-sectional survey

| **region** | **Village** | **village code** | **no. positive** | **N** | ***P.falciparum* prevalence** | **Prop.test (χ2)** | **p value** |
| --- | --- | --- | --- | --- | --- | --- | --- |
| 1 | Bessi | A | 5 | 308 | 1.6 | 9 | 0.003 |
|  | Ndemban Tenda | B | 11 | 141 | 7.8 |  |  |
| 2 | Chogen Wellingara | C | 21 | 205 | 10.2 | 14.9 | 0 |
|  | Yallal Ba | D | 6 | 302 | 1.9 |  |  |
| 3 | Dongoro Ba | F | 45 | 300 | 15 | 6 | 0.015 |
|  | Sinchu Njengudi | E | 5 | 100 | 5 |  |  |
| 4 | Sare Seedy | G | 17 | 321 | 5.3 | 3.1 | 0.08 |
|  | Ngedden | H | 3 | 180 | 1.7 |  |  |
| 5 | Njaiyel | J | 65 | 307 | 21.2 | 53.4 | 0 |
|  | Madina Samako | K | 169 | 345 | 48.8 |  |  |
| 6 | Sare Wuro | L | 69 | 247 | 27.9 | 9.6 | 0.002 |
|  | Gunjur Koto | M | 86 | 203 | 42.4 |  |  |

Data from Mwesigwa *et al*. On-going malaria transmission in The Gambia despite high coverage of control interventions: a nationwide cross-sectional survey. *Malar J*. 2015;14(1):314. Prop.test is paired test of proportions between villages in the same region

Table 2: Species specific mortalities to DDT and deltamethrin performed using Marascuilo procedure.

| Proportion pairs | Value (difference in proportions) | Critical range |  |
| --- | --- | --- | --- |
| **DDT** |  |  |  |
| *P1-P2* | 0.044 | 0.084 | Not significant |
| *P1-P3* | 0.545 | 0.137 | Significant |
| *P1-P4* | 0.085 | 0.069 | Significant |
| *P2-P3* | 0.589 | 0.099 | Significant |
| *P2-P4* | 0.041 | 0.038 | Significant |
| *P3-P4* | 0.63 | 0.427 | Significant |
|  |  |  |  |
| **Deltamethrin** |  |  |  |
| *P1-P2* | 0.05 | 0.097 | Not significant |
| *P1-P3* | 0.192 | 0.131 | Significant |
| *P1-P4* | 0.192 | 0.414 | Not significant |
| *P2-P3* | 0.242 | 0.109 | Significant |
| *P2-P4* | 0.243 | 0.408 | Not significant |
| *P3-P4* | 0.001 | 0.417 | Not significant |
| *P1*-*An. arabiensis*, *P2*- *An. coluzzii*, *P3*- *An. gambiae s.s., P4*- *An. coluzzii × An. gambiae s.s.* hybrid. The test statistic is the absolute difference between estimated mortality proportions. If value (pair wise difference in proportions) is higher than critical range, difference is considered significant. | | | |

**Table 3:** **Generalized Linear model testing the effects of village, species and kdr on mortality of mosquitoes to DDT**

| **Factors** | **Factor levels** | **Estimate** | **Std. Error** | **z.value** | **p** |
| --- | --- | --- | --- | --- | --- |
|  | (Intercept) | -3.9 | 0.86 | -4.53 | < 0.001 |
| Village | Yallal Ba | -0.79 | 0.99 | -0.79 | 0.428 |
|  | Sinchu Njengudi | 0.3 | 1.02 | 0.3 | 0.765 |
|  | Dongoro Ba | 0.95 | 0.95 | 1 | 0.319 |
|  | Sare Seedy | -17.12 | 1410.25 | -0.01 | 0.99 |
|  | Ngedden | -1.26 | 0.98 | -1.29 | 0.199 |
|  | Madina Samako | 1.8 | 0.85 | 2.11 | 0.035 |
|  | Sare Wuro | -2.09 | 1.39 | -1.51 | 0.132 |
|  | Gunjur Koto | 0.88 | 1.03 | 0.86 | 0.392 |
| Species | *An. coluzzii* | 1.36 | 0.77 | 1.76 | 0.079 |
|  | *An.col-An.gam* hybrid | -16.45 | 3063.43 | -0.01 | 0.996 |
|  | *An. gambiae s.s.* | 0.87 | 0.85 | 1.03 | 0.302 |
| *Kdr* | *Kdr: FF* | 5.77 | 1.13 | 5.1 | < 0.001 |
|  | *Kdr: FS* | 4.47 | 1.39 | 3.21 | < 0.01 |
|  | *Kdr: LF* | 2.08 | 0.89 | 2.33 | 0.02 |
|  | *Kdr: LS* | 1.63 | 0.86 | 1.88 | 0.06 |
|  | *Kdr: SS* | -15.99 | 7257.14 | 0 | 0.998 |
| *Kdr* refers to Knockdown resistance gene. Letters denote amino acid subsititutions at the 1014 codon; *FF*- homozygous for phenylalanine, *FS* has both phenylalanine and serine, *LF* – heterozygous for phenylalanine, *LS* – heterozygous for serine and *SS* – homozygous for serine | | | | | |

Table 4: Generalized Linear model testing the effects of village, species and kdr on mortality of mosquitoes to deltamethrin.

| Factor | Factor levels | Estimate | Std. Error | z.value | p |
| --- | --- | --- | --- | --- | --- |
|  | (Intercept) | -3.1 | 0.69 | -4.46 | < 0.001 |
| Village | Bessi | 1.36 | 0.66 | 2.05 | 0.041 |
|  | Yallal Ba | 0.97 | 0.65 | 1.5 | 0.135 |
|  | Dongoro Ba | 0.7 | 0.67 | 1.04 | 0.3 |
|  | Sare Seedy | 1.59 | 0.95 | 1.68 | 0.093 |
|  | Ngedden | 1.01 | 0.94 | 1.07 | 0.285 |
|  | Madina Samako | 1.94 | 0.95 | 2.04 | 0.042 |
|  | Sare Wuro | 0.47 | 0.97 | 0.48 | 0.631 |
|  | Gunjur Koto | -0.11 | 0.74 | -0.15 | 0.884 |
| Species | *An. coluzzii* | -0.89 | 0.77 | -1.16 | 0.246 |
|  | *An. col - An. gam s.s.*hybrid | 0.5 | 0.92 | 0.55 | 0.584 |
|  | *An. gambiae s.s.* | 0.28 | 0.55 | 0.52 | 0.603 |
| *kdr* | *Kdr: FF* | 2.99 | 0.68 | 4.42 | < 0.001 |
|  | *Kdr: FS* | -12.43 | 882.74 | -0.01 | 0.989 |
|  | *Kdr: LF* | 1.8 | 0.53 | 3.4 | < 0.001 |
|  | *Kdr: LS* | 0.06 | 0.86 | 0.07 | 0.947 |
|  | *Kdr: SS* | 2.52 | 1.52 | 1.65 | 0.098 |
| *Kdr* refers to Knockdown resistance gene. Letters denote amino acid substitutions at the 1014 codon; *FF*- homozygous for phenylalanine, *FS* has both phenylalanine and serine, *LF* – heterozygous for phenylalanine, *LS* – heterozygous for serine and *SS* – homozygous for serine | | | | | |
